# Interpretation of white blood cell counts in the cerebrospinal fluid of neonates with traumatic lumbar puncture: a retrospective cohort study

**DOI:** 10.1186/s12887-022-03548-z

**Published:** 2022-08-16

**Authors:** Gema García-De la Rosa, Silvia De las Heras-Flórez, Jorge Rodríguez-Afonso, Mercedes Carretero-Pérez

**Affiliations:** Department of Clinical Analysis Laboratory, Hospital Universitario Nuestra Señora de Candelaria, Carretera General del Rosario 145, 38010 Santa Cruz de Tenerife, Spain

**Keywords:** Bacterial meningitis, Cerebrospinal fluid, Newborn, Spinal puncture, Viral meningitis

## Abstract

**Background:**

Difficulty in interpreting white blood cell (WBC) counts in cerebrospinal fluid (CSF) complicates the diagnosis of neonatal meningitis in traumatic lumbar punctures (LP). The aim of our study was to determine the correction factor for WBC counts in traumatic LP that offers the greatest diagnostic efficacy in meningitis.

**Methods:**

We conducted a retrospective observational study of LP in neonates between January 2014 and December 2020. Traumatic LP was defined as a red blood cell (RBC) count ≥ 1,000 cells/mm^3^ CSF and pleocytosis as WBCs ≥ 20 cells/mm^3^ CSF. The CSF RBC:WBC ratio was analyzed by linear regression to determine a new correction factor. Cell count adjustments were also studied using the 500:1, the 1,000:1 ratio method, and the peripheral blood RBC:WBC ratio, using ROC curves and studies of accuracy (sensitivity and specificity).

**Results:**

Overall, 41.0% of the 1,053 LPs included in the study were traumatic. The best results for effective WBC correction were the method based on the peripheral blood ratio (sensitivity = 1.0 and specificity = 0.9 for bacterial meningitis and sensitivity = 0.8 and specificity = 0.9 for viral meningitis) and the 400:1 ratio (sensitivity = 1.0 and specificity = 0.8 for bacterial meningitis and sensitivity = 0.8 and specificity = 0.8 for viral meningitis) obtained from linear regression (95% CI 381.7–427.4; R2 = 0.7).

**Conclusion:**

Both the peripheral blood correction and the 400:1 correction reduce the number of neonates classified with pleocytosis who were not eventually diagnosed with meningitis. Both methods might be a useful tool to clarify the neonatal meningitis diagnosis, offering neonatologists the possibility to assess the WBC count in traumatic LP.

## Background

Meningitis occurs most often in the first month of life and is associated with high rates of morbidity and mortality [1–3]. The signs and symptoms of neonatal meningitis are often nonspecific, making it difficult to decide when to perform a lumbar puncture (LP) to confirm diagnosis. Some neonatologists choose to perform LP on all newborns with suspected sepsis, but others take a more selective approach and perform LP only in neonates with specific symptoms, positive blood cultures, or other suspicious findings. In our hospital guidelines, the indications to perform a LP follow the recommendations by the Committee on Fetus and Newborn [4], which include: 1) culture-positive bacteremia; 2) clinical course or laboratory data suggestive of sepsis; and 3) not show any clinical improvement with initial antimicrobial therapy, except those cases where they are forced to postpone the procedure due to cardiorespiratory instability [5]. Once a decision is made to perform LP, correctly interpreting the white blood cell (WBC) count in cerebrospinal fluid (CSF) and determining the presence of pleocytosis remain a challenge in the diagnosis of neonatal meningitis in the case of traumatic LP.

Traumatic LP occurs when the needle used to perform the procedure causes accidental bleeding in the subarachnoid space. It is estimated that about 30%-50% of neonatal CSF samples are contaminated with blood. The introduction of peripheral blood into the CSF complicates the interpretation of the results by increasing the WBC count, due to the higher concentration of WBC in the plasma compared to CSF [1–3, 5–7]. If pleocytosis is seen in LP, neonatology physicians prescribe antibiotic treatment indicated for suspected meningitis. This decision becomes more complicated when the LP is traumatic, as cell counts are difficult to interpret; these patients are also generally treated for meningitis until confirmation of the diagnosis [8]. The consequences of this approach range from complications caused by the often unnecessary use of antibiotics to a longer hospital stay [1, 2, 9].

One possible way of obtaining a correct interpretation of CSF WBC values in case of traumatic LP is to apply correction factors using the CSF RBC count [5]. Correcting WBC levels according to RBC levels in CSF helps avoid the routine initiation of antibiotics in most neonates with traumatic LP by identifying neonates that can be safely managed without antibiotics pending diagnostic confirmation. However, consensus has not yet been reached, and the different correction factors proposed in the recent literature on meningitis remain controversial. For this reason, our main objective was to evaluate the diagnostic efficacy of correcting WBC counts in traumatic LPs using methods previously described in the literature and a correction calculated in our population to determine the correction factor with the greatest diagnostic efficiency, in order to provide guidelines for the correct interpretation of these levels in the case of traumatic LP in neonates.

## Methods

### Study population

We performed a retrospective observational study of lumbar punctures performed on neonates (≤ 28 days) admitted to the neonatology unit of the Hospital Universitario Nuestra Señora de Candelaria between January 1, 2014 and December 31, 2020 who underwent LP with subsequent cell count, PCR for viruses and bacteria, and CSF culture to rule out bacterial or viral meningitis in the context of neonatal sepsis. The study was approved by the ethical committee of the Complejo Hospitalario Universitario de Canarias (CHUNSC_2020_104).

CSFs for which cell counts were unavailable or that had coagulated were excluded. In cases in which several CSF samples were collected from the neonate during admission, the first was selected for the study.

General patient data (registration number, sex, weight, gestational age at birth, and neonatal age at which LP was performed) and diagnosis at discharge (bacterial or viral meningitis as defined in the following section) were collected from the DragoAE Hospital Information System.

Blood parameters (peripheral RBC and WBC), CSF laboratory parameters [RBC count (cells/mm^3^), WBC (cells/mm^3^), glucose (mmol/L), and proteins (g/L)], and microbiological results in CSF (cultures with identified microorganism and PCR for bacteria and viruses) and peripheral blood (cultures with identified microorganism) were obtained from the OpenLab Laboratory Computer System (LCS). The gold standard visual method, Neubauer’s chamber, was used for cell counting. CSF biochemical parameters were analyzed using the Dimension RxL Max (Siemens Inc., Marburg, Germany) automated system. Blood parameters were obtained using the Sysmex XN automatic analyzer (Sysmex Corporation Inc., Kobe, Japan). Samples for blood culture were received in BACT/ALERT® Culture Media (bioMérieux Inc., Marcy-l'Étoile, France). As these are pediatric blood cultures, only a single sample (1–5 mL) was required, which was incubated in the BACT/ALERT® 3D for a standardized period of 5 days. In case of tested positive, subcultures were carried out in blood, chocolate and Schaedler agar, by seeding by discharge or isolation. Samples for CSF culture were received in polypropylene sterile tubes and were sown with a calibrated loop for isolation in blood, chocolate and McConkey agar and in fluid thioglycollate medium. Those were incubated at 37ºC for 24–48 h in aerobiosis except chocolate agar which was incubated in chamber at 5–10% CO_2_. The FilmArray meningitis panel (Biomerieux Inc., Marcy-l'Étoile, France) that analyzes the most common pathogens causing meningitis, including the following viruses, bacteria and yeasts, was used to identify the causative pathogen: *Escherichia coli K1, Haemophilus influenzae, Listeria monocytogenes, Neisseria meningitidis, Streptococcus agalactiae, Streptococcus pneumoniae, Cytomegalovirus* (CMV), *Enterovirus, Herpes simplex virus 1* (HSV-1), *Herpes simplex virus 2* (HSV-2), *Human herpesvirus 6* (HHV-6), *Human parechovirus, Varicella zoster virus* (VZV) and *Cryptococcus neoformans/gatti.*

### Variables analyzed

Traumatic LP was defined as an RBC count in CSF greater than or equal to 1,000 cells/mm^3^ [6, 10]. CSF pleocytosis was defined as a WBC count in CSF greater than or equal to 20 cells/mm^3^ [11, 12].

According to data collected from medical records, bacterial meningitis was defined as: (1) diagnosis confirmed by the presence of clinical manifestations and the detection of pathogenic microorganisms in CSF by culture or molecular techniques or (2) high index of suspicion of diagnosis due to in CSF changes suggestive of a bacterial process (WBC > 20 cells/mm^3^, proteins > 1.5 g/L in preterm infants or > 1.0 g/L in term infants, glucose < 1.1 mmol/L in preterm infants, < 1.7 mmol/L in term infants or less than 50% in peripheral blood) and/or the detection of a bacteria known to cause bacterial central nervous system infection in positive blood culture when in the opinion of the clinician the patient had clear clinical manifestations. Viral meningitis was defined as a CSF PCR finding of viruses causing meningitis in neonates.

### Statistical analysis

The CSF RBC:WBC ratio was analyzed by linear regression, with the CSF RBC count as independent variable and CSF WBC count as dependent variable, to establish a new correction factor. WBC count adjustments were also studied using other correction factors: 500 RBC for 1 WBC (500:1), and 1000 RBC for 1 WBC (1000:1). We also used the correction factor based on the peripheral blood RBC:WBC ratio (corrected WBC = CSF WBCs – (CSF RBCs * peripheral blood WBCs / peripheral blood RBCs). These 4 proposed correction factors were analyzed using traumatic LP ROC curves. The area under the curve was also determined to evaluate their discriminative diagnostic power. Sensitivity, specificity, positive and negative likelihood ratios and positive and negative predictive values were assessed for the defined CSF pleocytosis cut-off (20 cells/mm^3^). In order to compare these corrections with the diagnostic efficacy in non-traumatic LP, the statistical study was also carried out in this cohort.

The SPPS statistical package (version 25.0) was used for data analysis.

## Results

### Study patient characteristics

**Fig. 1:**
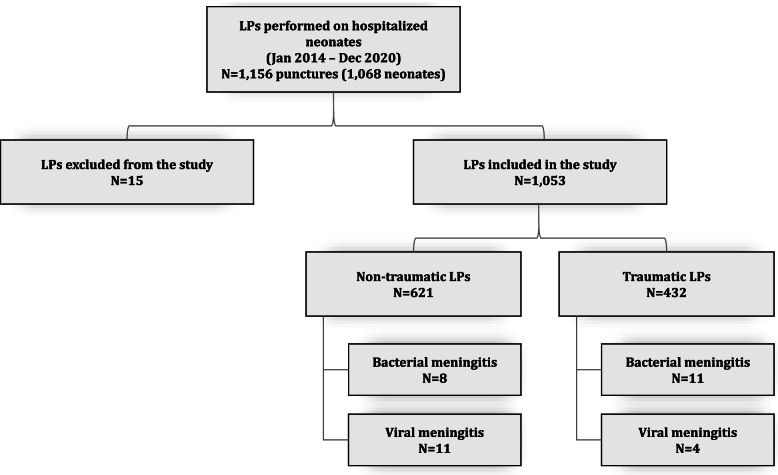
Flowchart of enrollment

A total of 1,156 LPs were performed on 1,068 neonates; only the first LP of neonates who underwent multiple LPs during admission was included in the analysis. Fifteen coagulated LPs were excluded due to the impossibility of performing a cell count. Of the 1,053 LPs included in the study, 41.0% (432) were traumatic (Fig. 1).

Among neonates with traumatic LPs, 2.5% (11/432) had bacterial meningitis and 0.9% (4/432) had viral meningitis. Of the remaining neonates with non-traumatic LPs, 1.3% (8/621) had bacterial meningitis and 1.8% (11/621) had viral meningitis (Fig. 1). With respect to the neonates classified into bacterial meningitis, 9 of them were categorized into diagnosis confirmed and the remaining 10 into high index of suspicion of diagnosis. We identified the following pathogenic microorganisms in neonates: *E. coli* (5), *Enterovirus* (15), *S. agalactiae* (2), *L. monocytogenes* (1) and *Klebsiella pneumoniae (1).* Fourteen of the 15 neonates diagnosed with viral meningitis were treated with antibiotics until diagnostic confirmation.

There was no statistically significant difference in demographic data and meningitis diagnoses between non-traumatic and traumatic LPs, except for neonatal age which was slightly higher in the non-traumatic LPs cohort (*p* = 0,005) (Table 1).

**Table 1:** Comparison of demographic, diagnostic data and characteristics of CSF in neonates with non-traumatic LPs and traumatic LPs

	**NON-TRAUMATIC LPs (*N* = 621)**	**TRAUMATIC LPs** **(*N* = 432)**	***P***
**Demographic data**			
Gestational age (weeks), median (IQR)	40 (39–41)	40 (38–40.5)	0.065^*^
Neonatal age (days), median (IQR)	1 (1–3)	1 (1–2)	0.005^*^
Prematurity (n), (%)	67 (10.9%)	64 (14.8%)	0.050^†^
Weight (grams), medium (IQR)	3,340.5 (2,922.5–3,668.0)	3,301.0 (2,900.5–3,675.0)	0.462^*^
Sex (n male), (% male)	389 (62.6%)	261 (60.6%)	0.513^†^
**Final diagnosis**			
Bacterial meningitis (n), (%)	8 (1.3%)	11 (2.5%)	0.131^†^
Viral meningitis (n), (%)	11 (1.8%)	4 (0.9%)	0.255^†^
**CSF parameters**			
RBC (cells/mm^3^), median (IQR)	37.0 (5.0–193.5)	12,093.5 (3,452.8–41,800.0)	< 0.001^*^
WBC (cells/mm^3^), median (IQR)	7.0 (2.0–13.0)	30.0 (12.0–82.8)	< 0.001^*^

Abbreviations: *IQR* interquartile range

^*^ is Mann–Whitney test

^†^ is Chi-squared test

### Main results

The median [interquartile range (IQR)] CSF RBC count was 12,093.5 [3,452.8–41,800.0] cells/mm^3^ for neonates with traumatic LPs and 37.0 [5.0–193.5] cells/mm^3^ for neonates with non-traumatic LPs. The median CSF WBC count was 30.0 [12.0–82.8] cells/mm^3^ for neonates with traumatic LPs and 7.0 [2.0–13.0] cells/mm^3^ for neonates with non-traumatic LPs (Table 1). Both RBC and WBC parameters were statistically higher in the traumatic LP cohort (*p* < 0,001).

**Fig. 2:**
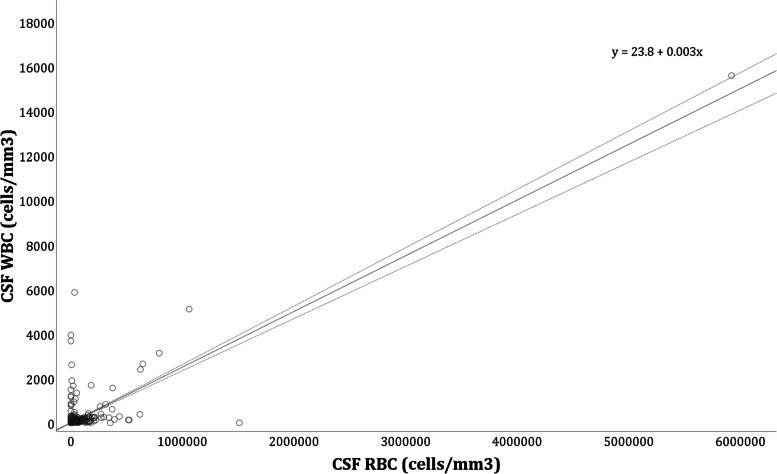
Relationship between CSF WBC and RBC count represented by linear regression (solid line) and its 95% confidence limits (dashed lines)

A CSF RBC:WBC correction ratio of 400:1 was estimated by linear regression (95% CI 381.7–427.4; R2 = 0.7) (Fig. 2).

### Correction model study

**Fig. 3:**
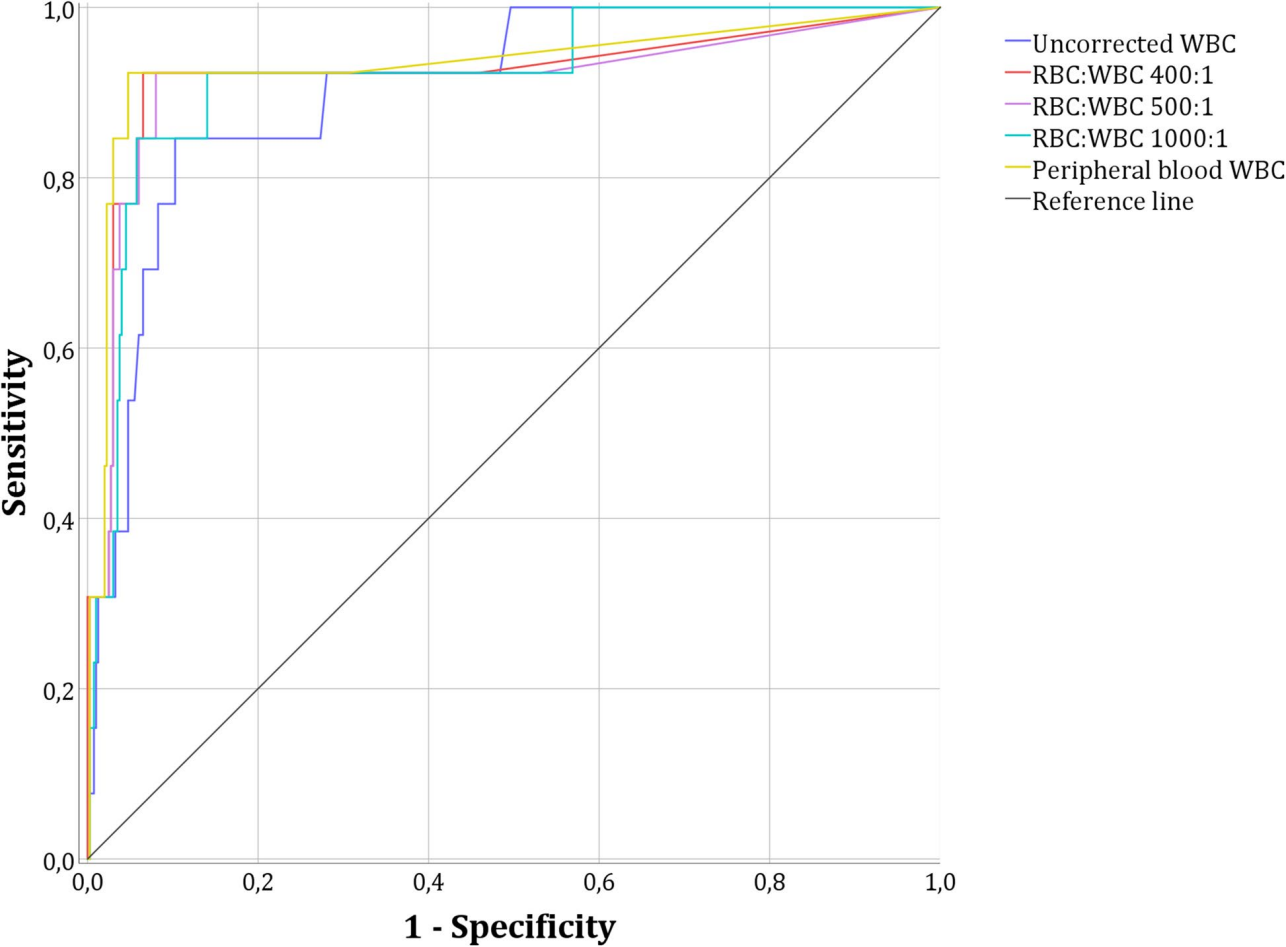
Comparison of the different CSF WBC correction formulas

The results of the comparison of the effectiveness of WBC correction in traumatic CSF using the factors 500:1, 1000:1 and 400:1 (the latter previously determined by linear regression), and the proportion of peripheral blood RBCs to WBCs in determining meningitis (bacterial and/or viral) are shown in Table 2 and Fig. 3.

**Table 2:** Comparison of different correction formulas for the detection of bacterial and/or viral meningitis in neonates with traumatic LPs

		**AUC (95% CI)**	**S**	**Sp**	** + LH**	**-LH**	**PPV**	**NPV**
**MENINGITIS**	**Uncorrected WBC**	0.91 (0.83–0.98)	1.00	0.35	1.54	0.00	0.06	1.00
	**RBC:WBC 500:1**	0.92 (0.81–1.00)	0.93	0.81	4.87	0.08	0.15	1.00
	**RBC:WBC 1000:1**	0.92 (0.84–1.00)	0.93	0.64	2.58	0.11	0.09	1.00
	**RBC:WBC 400:1**	0.92 (0.82–1.00)	0.93	0.84	5.99	0.08	0.18	1.00
	**Peripheral blood WBC**	0.93 (0.84–1.00)	0.92	0.90	9.44	0.09	0.24	1.00
**BACTERIAL** **MENINGITIS**	**Uncorrected WBC**	0.93 (0.87–0.98)	1.00	0.38	1.60	0.00	0.04	1.00
	**RBC:WBC 500:1**	0.97 (0.94–0.99)	1.00	0.80	5.07	0.00	0.12	1.00
	**RBC:WBC 1000:1**	0.96 (0.93–0.99)	1.00	0.63	2.73	0.00	0.07	1.00
	**RBC:WBC 400:1**	0.97 (0.95–0.99)	1.00	0.84	6.19	0.00	0.14	1.00
	**Peripheral blood WBC**	0.98 (0.96–0.99)	1.00	0.90	9.56	0.00	0.18	1.00
**VIRAL** **MENINGITIS**	**Uncorrected WBC**	0.84 (0.64–1.00)	1.00	0.37	1.59	0.00	0.02	1.00
	**RBC:WBC 500:1**	0.79 (0.46–1.00)	0.75	0.79	3.53	0.32	0.03	1.00
	**RBC:WBC 1000:1**	0.83 (0.60–1.00)	0.75	0.62	1.98	0.40	0.02	1.00
	**RBC:WBC 400:1**	0.80 (0.49–1.00)	0.75	0.82	4.22	0.30	0.04	1.00
	**Peripheral blood WBC**	0.82 (0.53–1.00)	0.75	0.87	5.59	0.29	0.06	1.00

Abbreviations: *AUC* area under the curve, *CI* confidence interval, *LH* likelihood ratio, *NPV* negative predictive value, *PPV* positive predictive value, *S* sensitivity, *Sp* specificity

When compared with the uncorrected WBC count, different correction factors showed lower sensitivity but higher specificity and area under the curve for the detection of meningitis. With regards to bacterial meningitis, sensitivity did not differ from the uncorrected WBC count (100%), although in regards to viral meningitis, correction factors showed lower sensitivity (75%).

The peripheral blood correction and 400:1 correction showed the highest specificity values for bacterial and viral meningitis (90% and 84% for meningitis, 90% and 84% for bacterial meningitis and 87% and 82% for viral meningitis, respectively) in comparison with the uncorrected WBC count (35% for meningitis, 38% for bacterial meningitis and 37% for viral meningitis). With these two methods, the peripheral blood correction and 400:1 correction, the number of neonates classified with CSF pleocytosis is substantially reduced: zero cases of bacterial meningitis and one single case of viral meningitis were misclassified.

Diagnostic efficacy was also evaluated in CSF with non-traumatic LPs. The results are summarized in Table 3. Compared with corrected traumatic LPs, the sensitivity in non-traumatic LPs was lower for meningitis and bacterial meningitis (84% and 88%, respectively) and the specificity was similar (88%, 87% and 87% for the detection of meningitis, bacterial meningitis and viral meningitis, respectively in non-traumatic LPs).

**Table 3:** Comparison of the effectiveness using WBC counts for the detection of bacterial and/or viral meningitis in neonates with non-traumatic LPs

	**AUC (95% CI)**	**S**	**Sp**	** + LH**	**-LH**	**PPV**	**NPV**
**MENINGITIS**	0.91 (0.81–1.00)	0.84	0.88	7.24	0.18	0.19	0.99
**BACTERIAL MENINGITIS**	0.94 (0.83–1.00)	0.88	0.87	6.79	0.14	0.08	1.00
**VIRAL MENINGITIS**	0.87 (0.73–1.00)	0.82	0.87	6.48	0.21	0.11	1.00

Abbreviations: *AUC* area under the curve, *CI* confidence interval, *LH* likelihood ratio, *NPV* negative predictive value, *PPV* positive predictive value, *S* sensitivity, *Sp* specificity

## Discussion

The high rate of traumatic LPs in neonates complicates clinical decisions surrounding the risk of both bacterial and viral meningitis [13]. Furthermore, consensus is lacking on the interpretation of cell counts in traumatic LPs and on the definition of traumatic LPs, with several CSF RBC cut-off points being proposed in the literature. For research purposes, the definition of traumatic LP ranges from 400 RBC/mm^3^ (the supposed visual threshold) to more than 10,000 RBC/mm^3^. However, the most widely used cut-off point used is 1,000 RBC/mm^3^ [9].

In our retrospective study of neonates undergoing LP over a period of 7 years, we observed a considerable number of traumatic LPs (41.0%), defined as ≥ 1,000 RBC/mm^3^. These data are consistent with studies reporting rates of up to 46% [5, 9], although comparison with other studies is complicated by the different definitions of traumatic LP used and by the various age ranges studied.

Baziomo et al. [14] suggest that the frequency of traumatic LP increases according to several factors, including the degree of prematurity. Our data are in line with this study and show an increase in the proportion of preterm infants who underwent traumatic LP compared to non-traumatic LP. Furthermore, recent studies also show a higher incidence of traumatic LP in patients with a lower neonatal age [2, 11, 15], again in line with our results. We found no significant differences in the proportion of traumatic LPs according to sex and birth weight, also in accordance with the latest published studies [1, 2].

The differential diagnosis between bacterial and viral meningitis is often complex, especially in neonates where clinical examination is of limited value [16]. Most neonates with CSF pleocytosis require admission and usually complete 10 to 14 days of intravenous antibiotic treatment until bacterial etiology is excluded [17]. In our study, we found that the use of empirical antibiotics until diagnostic confirmation is also common in neonates who were finally diagnosed with viral meningitis, suggesting that viral meningitis in neonates is often clinically indistinguishable from bacterial meningitis and that WBC correction is equally useful in traumatic LPs.

When we compare the AUC of the different correction methods for WBC counts in CSF, we obtain higher figures with the corrections 400:1, 500:1, 1000:1, and with the correction based on peripheral blood than that obtained with uncorrected WBC. Moreover, specificity values for uncorrected WBCs were 38% and 37% in bacterial and viral meningitis, respectively, while these figures increased significantly with all correction factors applied. If we focus on corrections that are not based on ratios in peripheral blood, we see that, although the factors of 500:1 and 1000:1 are the most widely used in the literature, the factor 400:1 obtained the best results in terms of specificity (84% for bacterial meningitis and 82% for viral meningitis). However, the peripheral blood correction obtained even better results (90% and 87% specificity for bacterial and viral meningitis, respectively).

Although the peripheral blood correction factor obtained the greatest specificity, it is more difficult to apply because recent blood data are required and more complex mathematical calculations must be employed. On the other hand, although correction by the 400:1 factor yields a slightly lower specificity, this method only requires CSF data and the mathematical calculation is easier. The specificity of the 500:1 correction is slightly lower than that of the 400:1 correction and may also be recommended because neonatologists are more familiar with this method.

The two largest studies to date evaluating WBC correction in traumatic LP reach conflicting conclusions. Since all these authors, including us, used different traumatic LP cut-off points, comparisons between studies are more difficult to interpret. For example, Greenberg et al. [3], who define traumatic LP as an RBC count ≥ 500 cells/mm^3^, conclude that WBC correction in traumatic LP is not useful because it leads to an underestimation of the real number of WBC present in CSF. It may certainly result in missed cases of meningitis that can entail serious clinical consequences. However, they did not take into account the possible morbidity resulting from unnecessary use of antibiotics in patients, which we believe should be a consideration. Lyons et al. [11] obtained a correction factor for the diagnosis of bacterial meningitis that was larger than ours (1000:1). However, it should be noted that their study population comprised infants aged up to 60 days. In their study, the application of the correction factor significantly reduced the number of children with CSF pleocytosis who eventually were not diagnosed with bacterial meningitis, a finding that is consistent with our study.

When comparing our results in corrected traumatic LPs, we observed that sensitivity and specificity results barely differed from those obtained from non-traumatic LPs that represent optimal CSF conditions and maximum reachable sensitivity and specificity. This substantial reduction in false positives, in relation with uncorrected WBC in traumatic LPs, with no increase in false negatives (missed diagnosis of meningitis), regarding the optimal reachable CSF conditions (non-traumatic LPs), demonstrates that correcting the WBC in traumatic LPs is a valuable procedure that is feasible for use in daily practice.

Our study has some limitations. Firstly, it is a single-center study with a limited number of cases of bacterial and viral meningitis. Large-scale multicenter studies are required to evaluate the interpretation of WBC count in traumatic LPs. It should, however, be noted that this is the first study of these characteristics to be carried out in the Spanish population. Secondly, we were unable to determine whether the study neonates received antibiotics prior to the LP procedure, which would overestimate WBC correction. However, this is an increasingly unusual clinical practice. In most cases, empirical therapy is initiated after LP is performed, except in cases where LP is not possible in the first instance due to clinical instability of the patient. Despite this, our study also included the cohort of neonates with non-traumatic LP in order to make a more reliable comparison. Thirdly, we did not select an upper limit for CSF RBC count in our definition of traumatic LP, so we cannot ensure that samples are exclusively peripheral blood. Fourthly, clinical data for the study neonates were not considered in this study. Clinicians treating neonates in the context of bacterial or viral meningitis should take into account not only CSF WBC counts, but also other clinical and laboratory variables.

## Conclusions

Although CSF correction factors should be used with caution in neonates, we show that correcting WBC values improved diagnostic yield in both bacterial and viral meningitis. Specifically, we recommend the use of both peripheral blood data and the 400:1 correction, the latter being readily available and easier to use. With both methods, it is possible to substantially reduce the number of neonates classified with CSF pleocytosis in whom bacterial or viral meningitis are finally not diagnosed. This approach offers neonatologists the possibility to assess WBC in CSF from traumatic LPs and to avoid the systematic administration of antibiotics in all these neonates.

## Data Availability

The datasets generated and/or analysed during the current study are available in the Harvard Dataverse repository, https://dataverse.harvard.edu/dataset.xhtml?persistentId=doi:10.7910/DVN/BNS5PL

## Abbreviations

CSF: Cerebrospinal fluid
LP: Lumbar punctures
RBC: Red blood cell
WBC: White blood cell
IQR: Interquartile range
